# Toxic metabolites, MAPK and Nrf2/Keap1 signaling pathways involved in oxidative toxicity in mice liver after chronic exposure to Mequindox

**DOI:** 10.1038/srep41854

**Published:** 2017-02-03

**Authors:** Qianying Liu, Zhixin Lei, Anxiong Huang, Qinghua Wu, Shuyu Xie, Ihsan Awais, Menghong Dai, Xu Wang, Zonghui Yuan

**Affiliations:** 1 National Reference Laboratory of Veterinary Drug Residues (HZAU) and MAO Key Laboratory for Detection of Veterinary Drug Residues, Huazhong Agricultural University, Wuhan, Hubei 430070, China; 2 MOA Laboratory for Risk Assessment of Quality and Safety of Livestock and Poultry Products, Huazhong Agricultural University, Wuhan, Hubei 430070, China; 3 College of Life Science, Yangtze University, Jingzhou, China; 4 Department of Chemistry, Faculty of Science, University of Hradec Kralove, Hradec Kralove, Czech Republic; 5 Hubei Collaborative Innovation Center for Animal Nutrition and Feed Safety, Wuhan, Hubei, China; 6 Department of Biosciences, COMSATS Institute of Information Technology, Sahiwal, Pakistan

## Abstract

Mequindox (MEQ) is a synthetic antimicrobial agent of quinoxaline-1,4-dioxide group (QdNOs). The liver is regarded as the toxicity target of QdNOs, and the role of *N* → O group-associated various toxicities mediated by QdNOs is well recognized. However, the mechanism underlying the *in vivo* effects of MEQ on the liver, and whether the metabolic pathway of MEQ is altered in response to the pathophysiological conditions still remain unclear. We now provide evidence that MEQ triggers oxidative damage in the liver. Moreover, using LC/MS-ITTOF analysis, two metabolites of MEQ were detected in the liver, which directly confirms the potential connection between *N* → O group reduction metabolism of MEQ and liver toxicity. The gender difference in MEQ-induced oxidative stress might be due to adrenal toxicity and the generation of M4 (2-isoethanol 1-desoxymequindox). Furthermore, up-regulation of the MAPK and Nrf2-Keap1 family and phase II detoxifying enzymes (HO-1, GCLC and NQO1) were also observed. The present study demonstrated for the first time the protein peroxidation and a proposal metabolic pathway after chronic exposure of MEQ, and illustrated that the MAPK, Nrf2-Keap1 and NF-кB signaling pathways, as well as the altered metabolism of MEQ, were involved in oxidative toxicity mediated by MEQ *in vivo*.

Quinoxaline-di-*N*-oxides (QdNOs) possessing the quinoxaline-1,4-dioxide basic structure, are known to be potent antibacterial agents with a wide range of biological properties^1^,^2^,^3^,^4^,^5^,^6^,^7^. They had been developed for use in livestock because of their significant antibacterial abilities and growth-promoting properties^3^,^6^,^7^,^8^,^9^,^10^. Carbadox (CBX), olaquindox (OLA) and quinocetone (QCT) are the members of QdNOs^5^,^6^. Mequindox (3-methyl-2-acetyl-*N*-1,4-dioxyquinoxaline, C_11_H_10_N_2_O_3_; MEQ) (Fig. 1), acting as inhibitor of DNA synthesis, is a synthetic QdNOs which acts as an antibacterial agent^11^,^12^,^13^. MEQ has been widely applied in pigs and chickens in China since the 1980s owing to its strong inhibitory activities against both gram-positive and -negative species^14^.

A lot of evidence suggested that the major metabolic pathway for QdNOs involves *N* → O group reduction^1^,^2^,^15^, and that this type of metabolism is closely related to their toxicity^10^,^16^,^17^. QdNOs were reported to have the ability of hypoxia–selective DNA cleavage^2^,^18^. A few studies revealed that the metabolites of CBX, OLA and QCT exhibit varying degrees of toxicity^5^,^10^,^19^. 2-isoethanol 4-desoxymequindox (M11), an *N* → O reduction metabolite of MEQ, was detected in the testis of the Wistar rats after the exposure of MEQ (25–275 mg/kg diet) for 180 days, where it was accompanied by oxidative DNA damage^12^. MEQ and its primary metabolites *N*1-desoxymequindox (*N*1-MEQ) and bidesoxy-mequindox (B-MEQ) (Fig. 1), were also found to be genotoxic in short-term *in vitro* and *in vivo* tests^2^,^20^. Therefore, the metabolites were thought to participate in MEQ–induced liver damage. However, to our knowledge, only two published studies have examined the effects of MEQ metabolites on the liver *in vivo*. One study aimed to identify the metabolism, distribution, and elimination of MEQ in pigs, chickens, and rats^14^, and another provided evidence that the metabolites of MEQ were related to the oxidative damage observed in Wistar rats^21^. It has been well established that there are generally species-specific differences in the metabolism of drugs, and the expression of drug-metabolizing enzymes may be altered by pathophysiological conditions, including oxidative stress. The metabolism of MEQ may also be altered *in vivo* after chronic exposure of animals. Therefore, it still needs to determine the potential toxic metabolites and the possible metabolic pathway of MEQ in the mouse liver under oxidative toxicity in the chronic toxic study of MEQ.

In previous studies, oxidative stress was found to be closely related to the damaging effects of QdNOs, such as apoptosis, DNA and lipid damage *in vivo* and *in vitro*^22^,^23^,^24^. MEQ exhibited mutagenicity and was a potent inducer of DNA damage via the production of reactive oxygen species(ROS)^25^. Oxidative-stress was also involved in the genotoxicity induced by QCT and OLA^26^,^27^. As for the mechanism of genotoxicity, the production of ROS, including superoxide anion (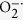), hydroxyl radicals (HO^·^) and hydrogen peroxide (H_2_O_2_) during *N* → O reduction of QCT by xanthine oxidoreductase (XOR), was shown to be a main factor in the DNA strand breakage and generation of 8-hydroxy deoxyguanosine (8-OHdG)^5^. We recently showed that MEQ could result in adrenal toxicity by oxidative stress in H295R cells that originated from a human adrenocortical carcinoma^28^. It has been demonstrated that oxidative stress was one of the most important toxicity mechanisms in the adrenal gland^29^, liver and spleen^21^, and endocrine and reproductive systems^12^ in male Wistar rats following exposure to MEQ. Although the oxidative damage invoked by QdNOs, including MEQ, caused much more attentions, the role of some related signaling pathways still remains unclear.

The p38 mitogen-activated protein kinase (MAPK) and c-Jun N-terminal kinase (JNK) are members of MAPK cascades. They are activated by oxidative stress^30^,^31^,^32^,^33^,^34^,^35^ to adjust the intracellular redox status^36^. Nuclear factor κB (NF-κB) is a sequence-specific transcription factor that functions in the immunological and cellular detoxifying defense systems^34^,^37^. In response to oxidative stress, Nrf2 translocates into the nucleus after dissociating from Kelch-like ECH-associated protein 1 (Keap 1), and then binds to antioxidant response elements (AREs)^38^,^39^,^40^. The Nrf2/Keap1/ARE pathway is a major cellular defense mechanism against oxidative stress by mediating phase II detoxifying enzymes and antioxidant enzymes^41^. The p38-Nrf2 signaling pathway has been shown to modulate oxidative stress *in vitro*^42^,^43^ and *in vivo*^36^,^43^. Previous studies indicated that, along with ROS generation, the phosphorylation of p38 and JNK were significantly increased in HepG2 cells after exposure to OLA^44^ and QCT^45^. Additionally, the oxidative damage caused by QCT involved the over expression of Nrf2 in rat liver^46^,^47^ and H295R cells^48^. However, to date, it is not clear whether the activation of the MAPK, Nrf2-Keap1 and NF-κB pathways participate in oxidative liver damage caused by MEQ though they have the same quinoxaline ring.

Based on the above information, this study was designed to investigate the hypothesis that MEQ–induced liver toxicity was associated with oxidative stress, the metabolism of MEQ and the activation of the MAPKs, NF-κB, and Nrf2-Keap1 signaling pathways. Since the liver, which is highly vulnerable to oxidative stress due to its high metabolic rate and low levels of endogenous scavengers, has been identified as one of the main target organs of QdNOs^49^,^50^,^51^, we evaluated the liver toxicity in Kunming mice after exposure to MEQ for 11 months. In the present study, we investigated: (1) the effect of MEQ on body and liver weight, and morphological changes in livers; (2) the effect of MEQ on the activities of albumin (ALB), alkaline phosphatase (ALP), alanine aminotransferase (ALT) and aspartate aminotransferase (AST) in the serum of mice; (3) identification the metabolites of MEQ in liver by LC/MS-ITTOF analysis; (4) the effect of MEQ on the levels of reduced glutathione (GSH) and total superoxide dismutase (T-SOD), and on oxidative damage to lipids, proteins and DNA, as measured by the levels of malondialdehyde (MDA), protein carbonyl content (PCC), and 8-OHdG; (5) whether MEQ activated the oxidative stress upstream signaling pathways involved in liver damage (e.g. p38, JNK); (6) the effect of MEQ on the mRNA expression of some cytokines related to the activation and regulation of oxidative stress (eg. NF-кB, Nrf2, Keap1, GCLC, NQO1 and HO-1).

## Results

### Body weight and liver coefficients

**Figure 2:**
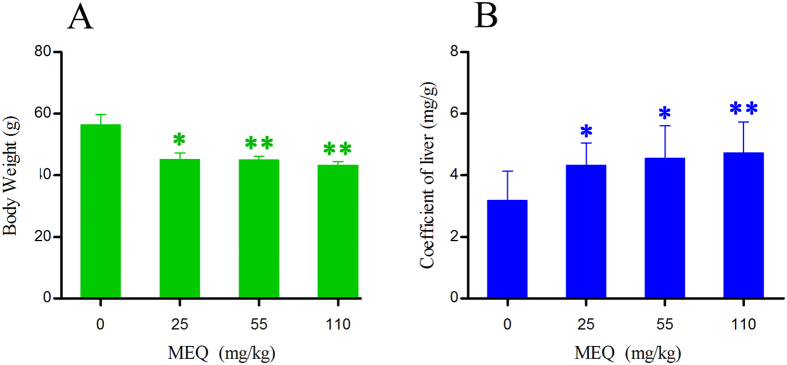
Body weight and liver coefficients of mice after administration of MEQ for 11 months. *p < 0.05, and **p < 0.01. Values represent means ± SD (n = 10).

The final body weight and liver coefficients of mice after administration of MEQ for 11 months are shown in Fig. 2. The liver coefficients was expressed as (wet weight of liver, mg)/(total body weight, g). Significant reductions in body weight were observed in the 25 mg/kg MEQ group (*p* < 0.05), and the 55, 110 mg/kg groups (*p* < 0.01) as compared with the control. Furthermore, there was a significant increase in the liver coefficients in the 25, 55 mg/kg MEQ (*p* < 0.05), and 110 mg/kg MEQ (*p* < 0.01) groups as compared with controls.

### Histopathological evaluation

**Figure 3:**
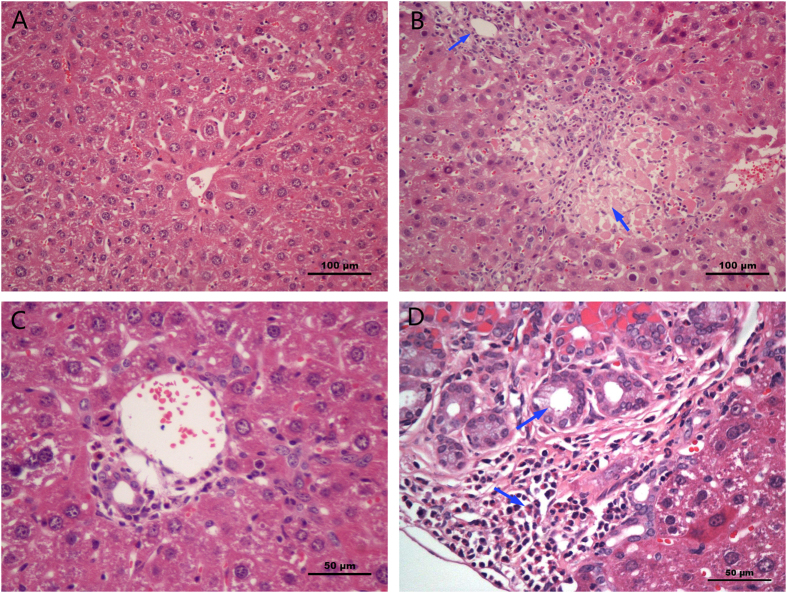
Selected microphotographs of liver (200×, 400×). (**A**) Control liver (200×). (**B**) Liver of the 110 mg/kg MEQ group showing degeneration and necrosis (200×). (**C**) Liver of the control group, the bile duct was normal (400×). (**D**) Liver of the 110 mg/kg MEQ group, the bile duct epithelium was marked with proliferation and neutrophil infiltration (400×).

As shown in Fig. 3, obvious histopathological changes were observed in the liver after administration of 55 and 110 mg/kg MEQ. The hepatic cells exhibited degeneration and necrosis (a necrotic lesion) in the 55 mg/kg MEQ group. In the 110 mg/kg MEQ group, there was marked neutrophilic infiltration within and around the bile duct and most bile duct epithelium examined showed proliferation, in contrast to control group.

### Hepatocellular enzymes and liver function indices

**Figure 4:**
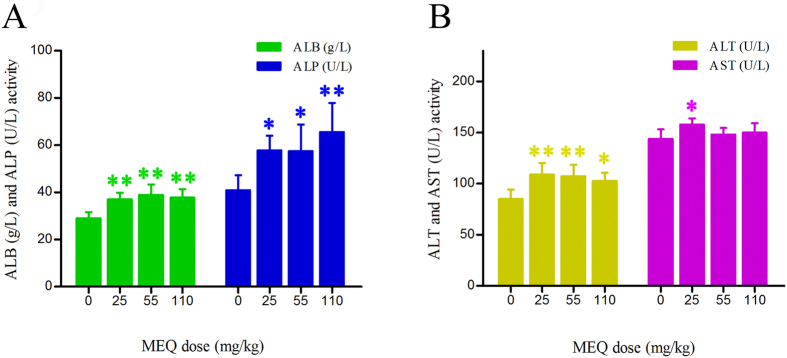
Effects of MEQ on the activity of ALB, ALP, ALT and AST in the mice serum after administration of MEQ for 11 months. *p < 0.05, and **p < 0.01. Values represent means ± SD (n = 10).

In comparison with the control group, three liver function indices – ALB, ALT and ALP – showed increased serum activity in all the MEQ–treated groups (Fig. 4).The 25 mg/kg MEQ group also showed significantly increased levels of AST as compared to controls (*p* < 0.05).This result was consistent with histopathological examination of liver, indicating the liver damage induced by MEQ.

### MEQ and its metabolites in liver

**Figure 5:**
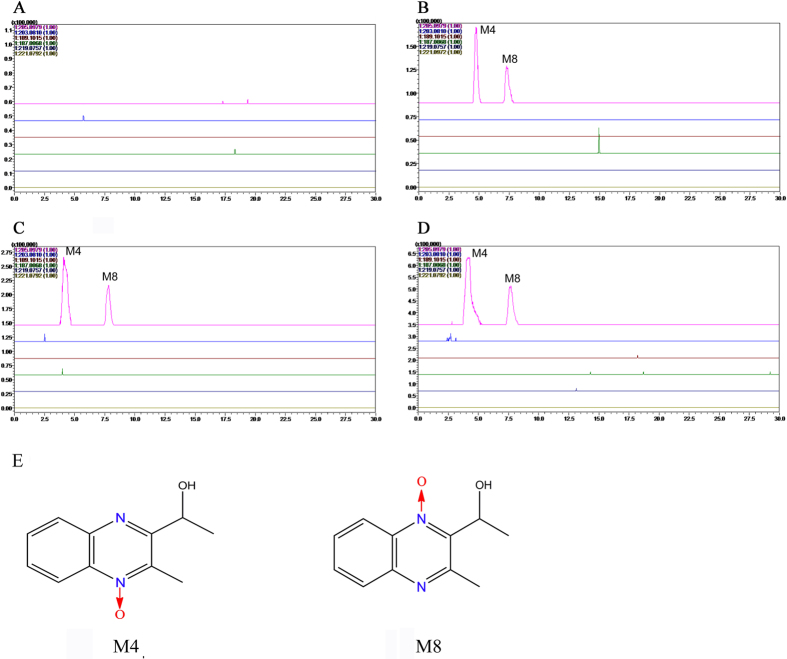
Accurate EIC of prototype and metabolites of MEQ in mice liver. (**A**) Liver of control; (**B**) Liver of 25 mg/kg MEQ diet; (**C**) Liver of 55 mg/kg MEQ diet; (**D**) Liver of 110 mg/kg MEQ diet; (**E**) The chemical structure of M4 is 2-isoethanol 1-desoxymequindox, and M8 is 2-isoethanol 4-desoxymequindox.

The prototype and metabolites of MEQ were determined according to the retention times and fragment ions. The results showed that noprototype of MEQ was found, while only two metabolites (M4 and M8)appeared in all treated groups (Fig. 5). Accurate MS^2^ spectra of M4 and M8 identified them as 2-isoethanol 1-desoxymequindox (the hydrogenation of the 1-desoxymequindox) and 2-isoethanol 4-desoxymequindox (the hydrogenation of the 4-desoxymequindox), respectively.

### Oxidative stress

**Figure 6:**
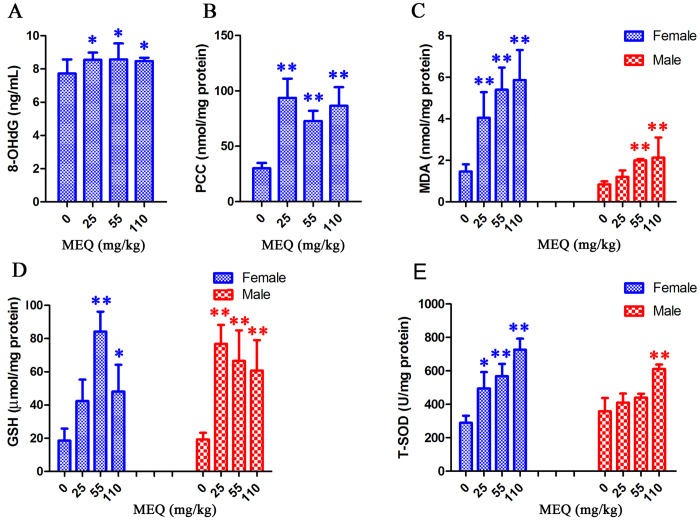
Effects of MEQ on (**A**) Peroxidation of DNA, (**B**) Protein carbonyl content, (**C**) Lipid peroxidation, (**D**) GSH, (**E**) T-SOD in mouse liver after administration of MEQ for 11 months. *p < 0.05, and **p < 0.01. Values represent means ± SD (n = 10).

To establish whether oxidative stress was elevated in the injured mouse liver, we measured the generation of T-SOD, GSH, MDA, PCC, and 8-OHdG in liver tissue as oxidative indices. As shown in Fig. 6, significant increases in the levels of 8-OHdG (*p* < 0.05) and PCC (*p* < 0.01) were noted in all treated groups as compared to controls. The levels of GSH, MDA and T-SOD were significantly higher in the 110 mg/kg than in controls (*p* < 0.01 or *p* < 0.05). Interestingly, there were significant gender differences in the levels of T-SOD, GSH and MDA induced by MEQ. At dose of 25 and 55 mg/kg MEQ, significantly increased levels of MDA and GSH were also observed in both genders (*p* < 0.01). However, after treatment with25 and 55 mg/kg MEQ, a significantly increased level of T-SOD was observed in females (*p* < 0.05, *p* < 0.01), but not in males. After treatment with MEQ, females showed higher levels of MDA and T-SOD as compared to males, while males showed higher levels of GSH as compared to females.

### Activation of the NF-кB, MAPK and Nrf2-Keap1 signaling pathways

**Figure 7:**
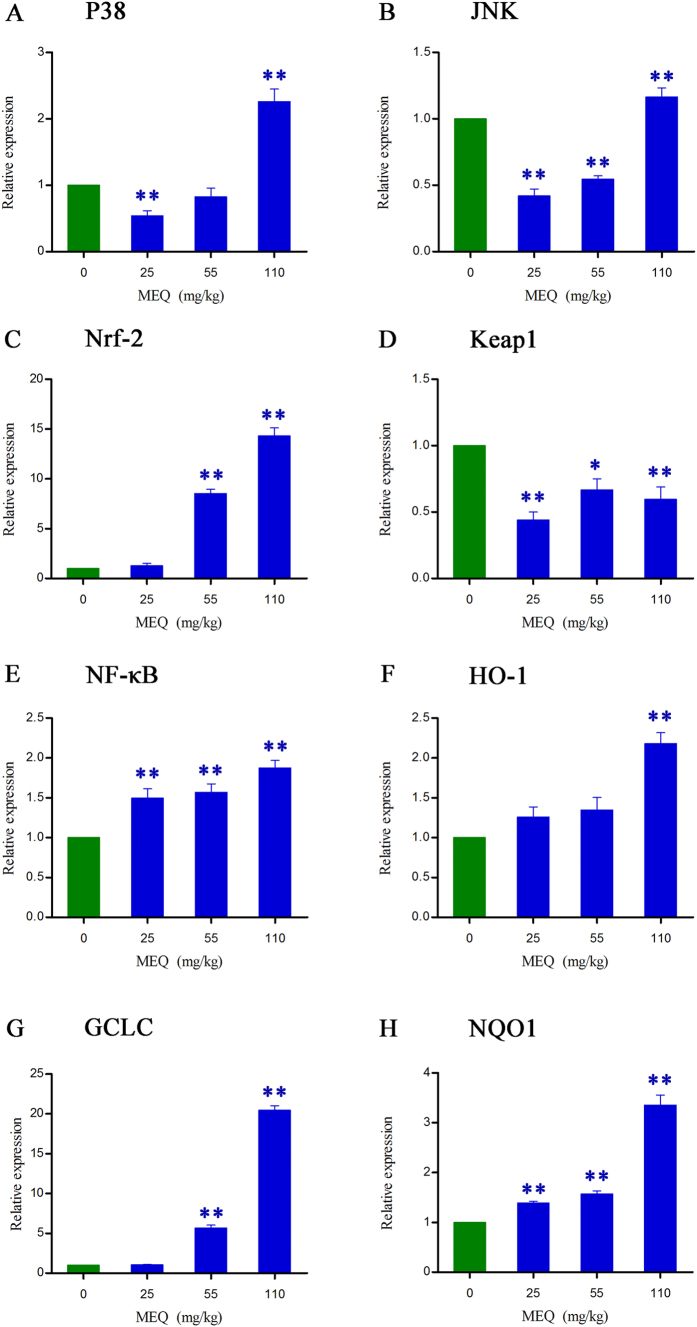
Alterations of P38, JNK, Nrf2, Keap1, NF-KB, HO-1, GCLC and NQO1 expression in mouse liver after administration of MEQ for 11 months. *p < 0.05, and **p < 0.01. Values represent means ± SD (n = 10).

To confirm whether MEQ–induced oxidative damage in the mouse liver induced various signaling pathways, the expression of some oxidative stress–related genes (e.g. p38, JNK, Nrf2, Keap1, NF-кB, HO-1, NQO1, GCLC) were evaluated using real-time quantitative RT-PCR (Fig. 7). Figure 7 shows that exposure to 110 mg/kg MEQ induced significant increases in p38 and JNK expression in the liver (*p* < 0.01). With increasing MEQ doses, there was a significant increase in Nrf2 expression (*p* < 0.01), and a marked reduction in Keap1 expression (*p* < 0.05 or *p* < 0.01). Exposure to MEQ significantly induced the expression of NF-кB and NQO1 in all the treated groups (*p* < 0.01). Increased mRNA expression of HO-1 was observed after treatment with 110 mg/kg MEQ (*p* < 0.01), and GCLC was significantly increased in both 55 and 110 mg/kg MEQgroups (*p* < 0.01). These results show a cellular protection response to oxidative stress mediated by chronic toxic study of MEQ.

## Discussion

Previous studies have demonstrated that oxidative stress is associated with the *in vitro* toxicity of QdNOs, including cytotoxicity^26^,^46^,^52^, adrenal toxicity^24^,^26^, genotoxicity^5^,^6^,^25^,^27^,^46^,^53^ and apoptosis^8^,^44^,^45^,^52^,^54^,^55^,^56^,^57^. *In vivo* studies in rats^11^,^12^,^21^,^29^,^47^,^58^,^59^ and mice^60^,^61^ have confirmed these findings. However, relatively little is known about the metabolism and the molecular mechanisms that mediate their toxicity. In the present study, we have demonstrated that the MAPK pathway, Nrf2-Keap1 family and NF-кB are closely related to the oxidative damage induced by MEQ in mouse liver. A higher sensitivity to oxidative damage was observed in females, as compared to males, after chronic administration of MEQ for up to 11 months. Furthermore, we identified that M4 and M8 were critical metabolites responsible for the liver toxicity induced by MEQ. Interestingly, the pathways of MEQ metabolism may be altered under a toxic environment via the altered expression of drug–metabolizing enzymes. Additionally, besides the traditional 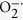 and HO^·^, the intermediate radicals of MEQ may be the key steps in triggering oxidative damage and activating the MAPK and Nrf2-Keap1 pathways.

Liver was identified as one of the main target organs for toxicity mediated by CYA^49^, QCT^50^ and MEQ^11^. MEQ induced disorganized hepatic cord patterning, cellular swelling and centrilobular liver cell necrosis in Wistar rats^11^. The altered levels of ALB, ALT, ALP and AST indicate chronic liver disease^11^,^49^. In the present study, a significant reduction in body weight and a significant increase in the liver coefficients were observed (Fig. 2). The histopathological evaluation showed marked liver damage, including extensive proliferation of the bile duct epithelium, and neutrophilic infiltrate within and around the bile duct. In biochemical analysis, MEQ–mediated increase in serum ALP exhibited disrupted plasma membrane bilayer and then resulted in efflux of serum ALT (cytosolic) and AST (mitochondrial). These results were consistent with previous reports suggesting that liver damage occurs after sub-chronic oral administration of MEQ in Wistar rats^11^,^21^,^29^.

**Figure 8:**
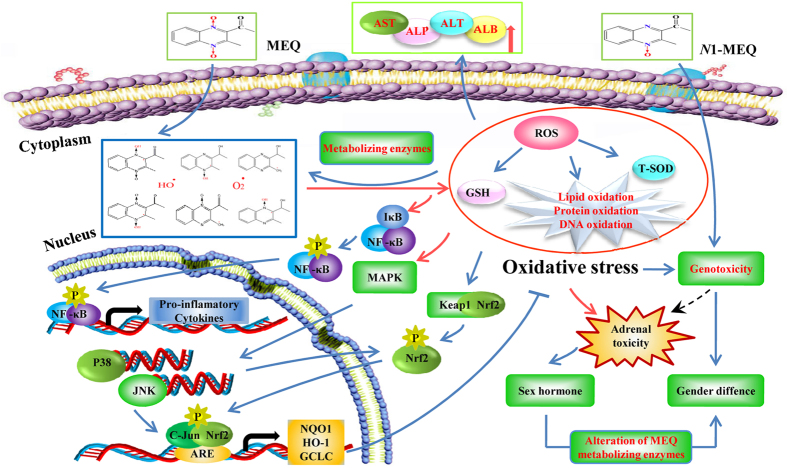
The proposed mechanisms of oxidative stress in mouse liver caused by MEQ. The oxidative damage occurs via the reduction of the *N* → O group of MEQ, which activate the MAPKs (e.g. P38 and JNK) signaling pathway, as well as Nrf2-Keap1 family, NF-κB and II phase detoxifying enzymes (e.g. HO-1, GCLC and NQO1). The sex difference in oxidative stress and genetoxicity caused by MEQ and *N*1-MEQ, respectively, may be derived from the adrenal toxicity along with the sex hormone secretion disorder.

A previous *in vitro* study reported that MEQ could be metabolized into ten metabolites after incubation with liver microsomes^62^. Recently, quantitative analysis of the *in vivo* metabolism of MEQ revealed that M4 was a common metabolite of MEQ in liver models of pigs, chickens and rats^14^. In another study of the relationship between the metabolites and toxicity, M11 was identified as the main toxic metabolite in the liver and spleen of Wistar rats after exposure to MEQ for 180 days^21^. The different metabolites detected in liver demonstrated the changed metabolic pathway of MEQ under the toxic environment, and therefore, M11 could be regarded as a biomarker of liver damage induced by MEQ. It is generally accepted that the metabolic activity of the enzymes such as aldo-keto reductases (AKR) and glutathione transferases (GST) has important consequences for the cellular redox status, and in turn, these enzymes could be regulated by oxidative stress^7^. Expression of drug–metabolizing enzymes may be altered in response to the pathophysiological conditions of the imbalance oxidative stress (Fig. 8). Thus, it was presumed that the alteration of MEQ–metabolizing enzymes such as carbonyl reductase 1 (CBR1), xanthine oxidoreductase (XOR), porcine aldehyde oxidase (SsAOX1) and cytochrome P450 (CYP)^34^ was possible a reason for the altered metabolic pathway of MEQ under liver damage status. Further study should focus on the expression and activity of these metabolizing enzymes after chronic exposure to MEQ. Herein, the detection of M4 and M8 not only directly confirmed the potential connection between *N* → O group reduction metabolism of MEQ and its organ toxicity, but also, indicated that these metabolites may act as biomarkers of liver toxicity in mice.

**Figure 9:**
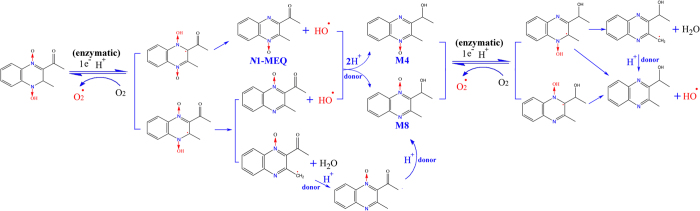
The proposed metabolic pathways of MEQ in mouse liver. The production of six MEQ intermediate radicals, as well as 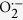 and OH^·^ occurs via the reduction of the *N* → O group of MEQ.

Apart from the deoxidation rate of QdNOs^6^, the appearance of ROS and unstable oxygen–sensitive radical intermediates during *N* → O reduction of QdNOs is considered to play a critical role in the DNA damage^1^,^18^,^63^,^64^. Previously, the production of oxygen–sensitive radical intermediates during QdNOs metabolism was thought to explain the mutagenicity^18^, antibacterial activity^1^ and hypoxic cytotoxin induced by these compounds^63^,^65^. The varying degrees of genotoxicity caused by QdNOs depends on the persistence of the ROS species^6^ and the stability of the radical intermediates^65^. Therefore, MEQ undergo intracellular one-electron enzymatic reduction to yield an oxygen–sensitive drug-radical intermediate and then generate highly reactive secondary radicals including 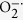, HO^·^ and MEQ radical intermediates that ultimately cause oxidative stress and DNA damage in the mouse liver (Figs 8 and 9). The metabolism of MEQ to M8 requires two steps, during which two radical intermediates were formed (Fig. 9). The proposed metabolic pathway in mice after chronic exposure of MEQ (Fig. 9) confirmed the critical role of radical intermediates in oxidative toxicity.

In order to demonstrate that exposure to MEQ caused oxidative stress in the mouse liver, we assayed five oxidative stress-related indicators. The results showed that MEQ caused significant increases in the levels of 8-OHdG, PCC, MDA, T-SOD and GSH. Previous studies also reported that the organ toxicity of MEQ *in vivo* was subjected to oxidative stress. In a study to investigate the metabolic response of mice after exposure to MEQ at 15, 75 and 350 mg/kg for 7 days, the oxidative damage was elevated by high doses of MEQ^61^. When rats were exposed to MEQ (25, 55, 110 and 275 mg/kg) for 180 days, high doses of MEQ caused testicular, liver, kidney,and adrenal toxicity, along with oxidative damage^12^,^21^,^29^. Oxidative stress could impair the antioxidant defense system and result in serious damage to cellular macromolecules such as DNA, lipids and protein^24^. However, to our knowledge, apart from MDA and 8-OHdG, no study about PCC induced by QdNOs was investigated. Here, we tested the ability of MEQ to induce protein peroxidation in the mouse liver, and we firstly found that protein was also one of the major targets of MEQ–induced oxidative damage.

As the most abundant intracellular antioxidant, SOD can serve as a redox biomarker of the antioxidant state^66^, and the role of GSH is to protect cells against oxidative damage^67^. It was reported that administration of MEQ (275 mg/kg diet) for 180 days in Wistar rats caused testicular, liver, kidney, and adrenal toxicity along with a significant increase in SOD activity and significant decrease in GSH levels^12^,^21^,^29^. The increased levels of T-SOD and GSH may be related to the activation of the upstream signaling pathway responsible for regulating oxidative stress. This hypothesis was confirmed by further study on the Nrf2-Keap1 family in mouse liver. Interestingly, there was an obvious sex difference in the levels of T-SOD, GSH and MDA. The levels of MDA and T-SOD were higher in females, while the activity of GSH was higher in males, suggesting more serious oxidative stress in females. These results were similar to a previous study that high doses of MEQ could significantly increase SOD activities in females when rats were treated with MEQ (55, 110 and 275 mg/kg) for 90 days^11^. Additionally, a few studies have reported adrenal toxicity caused by MEQ *in vivo*^26^,^29^ and *in vitro*^28^,^48^. Previous findings have shown that long-term MEQ treatment induced reproductive toxicity via alterations in the expression of some genes responsible for cholesterol transport and testosterone synthesis^12^, demonstrating the altered sex hormone secretion induced by MEQ. The changes in sex hormone secretion may be an indirect effect of adrenal toxicity induced by MEQ. These difference, in turn may explain the different sensitivities of males and females to MEQ – induced oxidative damage.

It was reported that the primary metabolites of MEQ – *N*1-MEQ and B-MEQ –were partially and completely reduced derivatives of MEQ, respectively^2^,^14^,^68^. Our recent research revealed that *N*1-MEQ showed higher adrenal toxicity than B-MEQ^28^. In amicronucleus test, the mice were treated with *N*1-MEQ at dose levels of 0.08, 0.16, 0.31 mg/kg, *N*1-MEQ (0.08 mg/kg) induced a significant increase in the ratio of micronucleated polychromatic erythrocytes (MN-PCEs) in females while not in males^2^, indicating an higher sensitivity of female mice to *N*1-MEQ-induced genotoxicity. The sex differences in *N*1-MEQ-induced genotoxicity may correspond to the levels of MEQ-induced oxidative stress (Fig. 8). Thus, we suspected that the occurrence of M4 (hydroxylation of *N*1-MEQ) may be another reason for the sex differences in MEQ–induced oxidative damage (Fig. 8).

To confirm whether the oxidative stress caused by MEQ is associated with the activation of some signaling pathway, we investigated MAPK signal transduction, which showed higher levels of p38 and JNK expression following exposure to MEQ (Fig. 7). In two studies investigating MAPK pathways in OLA–induced apoptosis^45^, and the molecular mechanisms of apoptosis induced by QCT^58^, a significant increase in p38 and JNK were observed. In the nucleus, Nrf2 first forms dimers with c-Jun before combining with ARE. The increased expression of JNK could activate c-Jun and indirectly stimulate the Nrf2-ARE pathway (Fig. 8). Moreover, after treatment with MEQ, the overexpressed of p38 and JNK in the plasma promote phosphorylation of Nrf2 (Fig. 8). NF-кB is a redox–sensitive transcription factor involved in the immunological and cellular detoxifying defense systems^36^,^59^,^69^. Previously, Yu *et al*. discovered that QCT-induced nephrotoxicity was accompanied by an increase in Nrf2 expression^47^, and the NF-кB and Nrf2/HO-1 pathways could protect cells against the oxidative stress induced by QCT^59^. The Nrf2-Keap1 signaling pathway plays a significant role in regulating the expression of phase II detoxifying enzymes and antioxidant enzymes that protect cells from endogenous and exogenous stresses^34^,^59^. Here, treatment with MEQ resulted in a significant increase in NF-кB and Nrf2 gene expression, and a significant decrease in Keap1 gene expression (Fig. 7). These findings indicated that the high level of p38, as well as JNK, NF-кB and Nrf2 expressions may be a cellular protective response to MEQ oxidative stress (Fig. 8).

To further reveal the possible downstream genes of Nrf2-Keap1 family following exposure to MEQ, we measured its target gene products including NQO1, HO-1 and GCLC. These enzymes protect against oxidative stress-induced toxicity either via scavenging ROS directly or via catalyzing the synthesis of antioxidant and detoxifying substrates indirectly^70^. Our data showed that MEQ exposure induced marked increases in HO-1 and GCLC levels (Fig. 7). These results indicated that HO-1 and GCLC may mediate a protective effect against MEQ–induced oxidative damage. NQO1 has been reported to play an important role in xenobiotic metabolism and in protecting against oxidative stress induced by the intermediates produced during xenobiotic detoxification^71^. Here, a significant dose-dependent increase in the induction of NQO1 was observed in all MEQ–treated groups. NQO1 may involved in the metabolic reaction of hydroxy and hydrogenated production of M4 and M8. In the present study, the increased expression of NQO1 was suggested to be associated with the generation of M4, M8 and MEQ radical intermediates. This result illustrates the important role of NQO1 in the metabolism of MEQ.

In conclusion, the current study demonstrated that M4 and M8 may represent the major toxic metabolites of MEQ causing oxidative liver damage *in vivo*, and that oxidative stress plays a key role in the liver toxicity. The pathways governing the metabolism of MEQ may be altered by changes in the activity of the MEQ–metabolizing enzymes under the imbalance of cellular redox status. Further study should be conducted to investigate the activity of the MEQ-metabolizing enzymes and the roles of MEQ radical intermediates under this case. Sex–specific differences in oxidative stress were observed in mice after administration of MEQ. It seems that the adrenal toxicity was possible an indirect reason for sex difference in oxidative stress caused by MEQ. The protein was firstly identified as the major target of MEQ–induced oxidative damage. The present findings also reveal that NF-кB, as well as MAPK (p38 and JNK) signaling pathways and the Nrf2-Keap1 family are involved in MEQ induced–redox imbalance damage in the mouse liver.

## Materials and Methods

### Chemical reagents

Mequindox (C_11_H_10_N_2_O_3_, molecular weight 218.21 g/mol, CAS No: 60875-16-3, purity 98%) was obtained from Beijing Zhongnongfa Pharmaceutical Co. Ltd. (Huanggang, PR China). MDA, PCC, SOD, GSH and 8-OHdG kits were obtained from Nanjing Jiancheng Bioengineering Institute (Nanjing, P.R. China). All other chemicals were purchased from Sigma (St. Louis, USA) unless otherwise stated.

### Animals and diets

Forty in total specific pathogen-free (SPF) Kunming mice (6–7 weeks old, weighting 25–36 g) were purchased from the Center of Laboratory Animals of Hubei Province (Wuhan, PR China). For each sex, the individual body weights were within ±20% of the average. The mice were maintained in a room conditioned at 22 ± 3 °C, a relative humidity of 50% ± 20%, and a 12 h light/dark cycle. In this study, the mice were handled in accordance with the guidelines and protocols approved by the Ethical Committee of the Faculty of Veterinary Medicine (Huazhong Agricultural University). Prior to initiation of dosing, mice were quarantined for 1 week to evaluate any signs of disease and weight gain. The mice received free access to fresh water and basic diet during the one week acclimatization period.

For the experiments, the mice were randomly divided into four groups (*N* = 10 per group), including a control group (received the basic diet without feed additives) and three treated groups (administrated the same diet supplemented with 25, 55 and 110 mg/kg MEQ). Mice were separated by sex and housed five per group in shoebox cages with hardwood shavings as bedding. Food and water were supplied *ad libitum* and the treatment period lasted for 11 months. Symptoms and/or mortality were observed and carefully recorded each day during the 11 month period. The use of animals in this study was in compliance with the NIH Publication “The Development of Science-Based Guidelines for Laboratory Animal Care”^72^.

### Coefficients and preparation of liver

Following 11 months of MEQ administration, all mice were subjected to fasting overnight. The mice were then weighted and sacrificed after being anesthetized with diethyl ether. After weighing the body and livers, the coefficient of liver was calculated as the ratio of liver (wet weight, mg) to body weight (BW) (g). The livers were excised, rinsed in phosphate buffered saline (PBS), weighed and then quickly frozen at −70 °C.

### Histopathological examination of liver

For pathological studies, all histopathological tests were performed using standard laboratory procedures. Half of livers were preserved in 10% neutral-buffered formalin. After fixation, the livers were embedded in paraffin blocks then sliced into 5 μm sections by microtome and placed onto glass slides for hematoxylin-eosin (HE) staining. Slides were observed under an optical microscope (Olympus BX 41, Japan) for morphological alterations.

### Biochemical analysis

For biochemical analysis, serum aliquots were obtained by placing the blood samples in serum tubes at a temperature of 24 °C for approximately 30 min. After clotting, the blood tubes were centrifuged at 3000 rpm for 10 min using a Himac CR 21 G centrifuge (Hitachi, Tokyo, Japan). Supernatants were removed and stored at −20 °C for further analysis. Serum chemistry was assessed using a Synchron CX4 Clinical System (Beckman Coulter, Brea, CA USA) according to the manufacturer’s protocol (Beijing Leadman Biochemistry Technology Co. Ltd, Beijing, China). The serum activities of ALB, ALP, ALT and AST were used as biochemical markers of hepatic damage.

### LC/MS-ITTOF analysis of MEQ and its metabolites in liver

The detection of mequindox and its metabolites in the liver was carried out using the hybrid IT/TOF mass spectrometer coupled with a high-performance liquid chromatography system (Shimadzu Corp., Kyoto, Japan). The liquid chromatography system (Shimadzu) was connected to a solvent delivery pump (LC-20AD), an autosampler (SIL-20AC), a DGU-20A3 degasser, a photodiode array detector (SPD-M20A), a communication base module (CBM-20A) and a column oven (CTO-20AC).

A total of 0.1 g of the liver sample was homogenized with 4.5 mL of 40 °C distilled water at the speed of 10,000 × g for 3 min in a model omni mixer homogenizer 17106 (OMNI International, Waterbury, CT, USA). Then 0.5 mL trichloroacetic acid was added at a final concentration of 10%. After vigorous shaking, the homogenate was centrifuged at 10,000 × g for 15 min to collect the supernatant. The mixed reagent [dichloromethane: acetonitrile (2:1, v/v)] was used to extract mequindox and its metabolites twice. Three milliliters of the mixed reagent was then added to the supernatant and vortex-mixed for 5 min. After vigorous shaking, the solution was centrifuged at 10,000 × g for 15 min. The lower liquid from the two extractions was merged and dried using N_2_ in a 40 °C water bath. The residue was reconstituted in 5 mL of 5% methanol. The reconstitution fluid was applied to a methanol (3 mL) and water (3 mL) pre-washed HLB 3cc cartridge (Waters Corporation, Milford, MA, USA). The reconstitution fluid was then sequentially washed with 3 mL of 5% methanol in water and 5 mL of methanol. The liver extracts were eluted into plastic tubes and evaporated to dryness under a nitrogen stream at 45 °C. Following drying, the residue was dissolved in 500 μL of LC-MS/MS mobile phase solution [acetonitrile: 0.1% formic acid (1:9, v/v)] and passed through a 0.22 μm filter membrane. The mixture (200 μL) was prepared for LC/MS-ITTOF analysis. Mequindox and its metabolites were identified based on a previous report^14^.

### Oxidative stress assay

Assays of MDA, T-SOD, GSH and PCC levels in liver were performed using commercial kits. 8-OHdG was assayed by using a commercial ELISA kit. Data were analyzed according to the manufacturer’s instructions. Protein concentration in liver tissue was measured using BCA protein assay kit.

### Expression of oxidative stress cytokines

The level of mRNA expression of oxidative stress-related genes, including p38, JNK, Nrf-2, Keap1, NF-кB, HO-1, GCLC, NQO-1, were determined by real-time quantitative reverse transcriptase-polymerase chain reaction (RT-PCR). Total RNA was isolated from the liver homogenates using the Trizol reagent according to the manufacturer’s instructions. One microgram of RNA was reverse transcribed to cDNA using the ReverTra Ace^TM^ First Strand cDNA Synthesis Kit (Promega, USA). Synthesized cDNA was used for quantitative real-time PCR (Bio-Rad, USA) by SYBR^®^ Premix Ex Taq^TM^ RT-PCR kit (Takara, CodeDRR041 A, Japan).

Mouse specific primers were designed using Primer Express Software according to the software guidelines (Table 1). Each 25 μL reaction mixture consisted of 12.5 μL SYBR^®^ Premix Ex Taq^TM^, 1.0 μL of each primer (10 μm), 2.0 μL of cDNA, and 8.5 μL Rnase Free H_2_O. For p38, JNK, HO-1, GCLC, the cycling conditions were as follows: step 1, 30 s at 95 °C; step 2, 45 cycles at 95 °C for 5 s, 55 °C for 30 s; step 3, dissociation stage. For Nrf-2, Keap1, NF-кB, NQO-1, the cycling conditions were as follows: step 1, 30 s at 95 °C; step 2, 45 cycles at 95 °C for 5 s, 60 °C for 30 s; step 3, dissociation stage. The housekeeping gene β-Actin was used as an internal calibrator reference gene for expression profiling of oxidative stress-related genes in this study.

Following amplification, a melting curve analysis was employed to verify the authenticity of the amplified product based on its specific melting temperature (Tm). The threshold cycle for the gene of interest and housekeeping gene, and the difference between their Ct values (ΔCt), were calculated. Relative quantitative analyses of gene expression were calculated using the 2^−ΔΔCt^ data analysis method in accordance with the previous literature^21^,^29^,^73^,^74^. The housekeeping gene β-Actin was employed for compression as a control.

### Statistical analysis

All results are expressed as mean ± SD. Statistical analysis was examined using the SPSS 15.0 software. Group differences were assessed by one-way analysis of variance followed by the least significance difference (LSD) test. *p* < 0.05 was considered statistically significant.

## Additional Information

**How to cite this article**: Liu, Q. *et al*. Toxic metabolites, MAPK and Nrf2/Keap1 signaling pathways involved in oxidative toxicity in mice liver after chronic exposure to Mequindox. *Sci. Rep.* **7**, 41854; doi: 10.1038/srep41854 (2017).

**Publisher's note:** Springer Nature remains neutral with regard to jurisdictional claims in published maps and institutional affiliations.

## Figures and Tables

**Figure 1:**
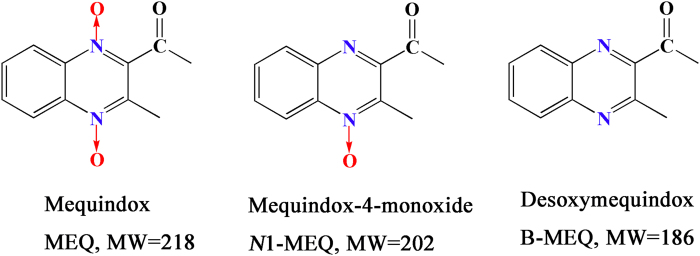
The chemical structures of mequindox (MEQ), mequindox-4-monoxide (*N*1-MEQ) and desoxymequindox (B-MEQ).

**Table 1:** PCR Primers Used in the Gene Expression Analysis.

Gene name	Description	Sequence (5′-3′)	Length (bps)
β-actin	mβ-actin - F	CTGTCCCTGTATGCCTCTG	221
	mβ-actin - R	TTGATGTCACGCACGATT	
P38	mP38 - F	GGAGAAGATGCTCGTTTTGGA	211
	mP38 - R	TTGGTCAAGGGGTGGTGG	
JNK	mJNK - F	TCTCCAGCACCCATACATCAA	150
	mJNK - R	TCCTCCAAATCCATTACCTCC	
Nrf-2	mNrf-2 - F	TCCTATGCGTGAATCCCAAT	103
	mNrf-2 - R	GCGGCTTGAATGTTTGTCTT	
Keap1	mKeap1 - F	GATCGGCTGCACTGAACTG	106
	mKeap1 - R	GGACTCGCAGCGTACGTT	
NF-κB	mNF-κB - F	GGTGGAGGCATGTTCGGTA	142
	mNF-κB - R	TGACCCCTGCGTTGGATT	
NQO1	mNQO1 - F	TTCTGTGGCTTCCAGGTCTT	104
	mNQO1 - R	TCCAGACGTTTCTTCCATCC	
GCLC	mGCLC - F	ATGTGGACACCCGATGCAGTATT	200
	mGCLC - R	GTCTTGCTTGTAGTCAGGATGGTTT	
HO-1	mHO-1 - F	GACAGAAGAGGCTAAGACCGC	213
	mHO-1 - R	TGGAGGAGCGGTGTCTGG	

Note: The primers were manufactured by Nanjing Genescript Co. Ltd. (Nanjing, PR China). P38, p38 MAP Kinase; JNK, c-Jun N-terminal protein kinase; Nrf-2, NF-E2-related factor 2; Keap1, Kelch-like ECH-associated protein1; NF-κB, nuclear factor κB; NQO1, NAD(P) H: quinoneoxidoreductase; HO-1, heme oxygenase 1; GCLC, catalytic subunit of glutamate-cysteine ligase.
